# Prolonged skin involvement distinguishes adolescent from childhood-onset IgA vasculitis: a large multicenter study with 687 patients

**DOI:** 10.1186/s42358-025-00516-w

**Published:** 2026-02-12

**Authors:** Luisa Fernanda Caro Forero, Paula Santana Marra, Ricardo Nobrega Machado, Clara Ribeiro Doria, Sebastian Durán Cordoba, Lia Vineyard Steuer, Matheus Santos França, Sylvia Costa Lima Farhat, Izabel Mantovani Buscatti, Gleice Clemente Souza Russo, Maria Teresa Terreri, Mayra Siqueira Dantas, Luciana M. Carvalho, Francisco H. R. Gomes, Natalia Maronese, Adriana R. Fonseca, Marta C. F. Rodrigues, Andreia Watanabe, Lucia M. A. Campos, Adriana Maluf Elias, Verena Andrade Balbi, Nadia Emi Aikawa, Vivianne S. L. Viana, Lara R. C. Melo, Claudia A. A. Strabelli, Magda Maria Sales Carneiro-Sampaio, Katia Tomie Kozu, Clovis Artur Silva

**Affiliations:** 1 Pediatric Rheumatology Unit, Instituto da Criança e do Adolescente, Hospital das Clinicas da Faculdade de Medicina da Universidade de São Paulo, Av. Dr. Enéas Carvalho de Aguiar, 647 - Cerqueira César, Sao Paulo, SP 05403-000 Brazil; 2 Pediatric Rheumatology Unit, Universidade Federal de Sao Paulo, Sao Paulo, Brazil; 3 Pediatric Rheumatology Unit, Ribeirao Preto Medical School, University of Sao Paulo, Ribeirao Preto, Brazil; 4 Rheumatology Division, Instituto de Puericultura e Pediatria Martagão Gesteira - Universidade Federal do Rio de Janeiro, Rio de Janeiro, Brazil

**Keywords:** IgA vasculitis, Henoch-Schönlein purpura, Adolescent, Children, Renal

## Abstract

**Background:**

Immunoglobulin A vasculitis (IgAV), also known as Henoch- Schönlein purpura (HSP), is the most common systemic vasculitis in childhood. Potential differences in demographic characteristics, clinical presentation, laboratory findings, and treatments approaches across age groups, remain poorly explored. To the best of our knowledge, no multicenter study in Latin America has systematically addressed these features. We aimed to assess demographic, clinical and laboratory features, and treatments in children *versus* adolescents with (IgAV)/ (HSP) in a large multicenter study.

**Methods:**

A multicenter study involving four tertiary centers evaluated 687 children and adolescents (≤ 18 years-old) with IgAV/HSP (EULAR/PRINTO/PRES classification criteria) at first 3 months after diagnosis. The charts were retrospectively assessed for demographic data, initial clinical manifestations, laboratory tests and treatments. Data were compared between children (< 10 years-old) and adolescents (≥ 10 years-old), according to WHO definition.

**Results:**

IgAV/HSP was diagnosed in 599/687(87%) children [5.33(0.88–9.91) years-old] and 88/687(13%) adolescents [11.33(10-17.5) years-old]. The median duration of purpura/petechiae was significantly lower in children compared to adolescents [14(1-120) vs. 15(2–90) days, *p* = 0.04]. The frequency of persistent purpura/petechiae (≥ 6 weeks of duration) was significantly reduced in the former group (7.2% vs. 19.5%, *p* = 0.002), likewise the frequency of gastrointestinal bleeding (17% vs. 34.1%, *p* = 0.01) and proteinuria (49.7% vs. 84%, *p* = 0.002). In contrast, the frequencies of arthritis/arthralgia (82.7% vs. 73%, *p* = 0.03) and orchitis(16.6% vs. 4.8%, *p* = 0.04) were significantly higher in children. Further analysis of laboratory tests showed that the median value of serum IgA was significantly lower in children than in adolescents [179.1(40-1002.0) vs. 279.0(104.0-488.0) mg/dL, *p* = 0.01], whereas thrombocytosis was higher (40.1% vs. 23%, *p* = 0.007). Logistic regression demonstrated that persistent purpura/petechiae after IgAV/HSP diagnosis (OR = 18.337; 95%CI 1.245-270.137; *p* = 0.034) was the only independently associated variable with dependent variable (adolescent).

**Conclusions:**

In this large multicenter cohort, IgAV/HSP onset occurred rarely at adolescence, with a more prominent cutaneous involvement. Prolonged purpura/petechiae after IgAV/HSP diagnosis was associated with adolescent-onset IgAV/HSP, reinforcing the need for vigilant monitoring in this subgroup.

## Background

Immunoglobulin A (IgA) vasculitis, formerly known as Henoch-Schönlein purpura (HSP), is the most common form of primary vasculitis observed in pediatric populations [1]. This systemic inflammatory condition may affect children and adolescents with involvement of the skin, joints, kidneys and gastrointestinal tract, and rarely testicles, central nervous system and lungs [2]. Median age at diagnosis of IgAV/HSP in different pediatric populations ranges from 6.0 to 10.2 years [1–9].

Comparisons of disease and treatment parameters among different pediatric age groups have not been studied in a large-scale population, particularly at the time of diagnosis. A recent single-center study from Turkey evaluating IgAV/HSP revealed that adolescents presented more severe disease manifestations compared to children at diagnosis. This report showed higher frequencies of joint and renal involvements, as well as an increased need for aggressive treatment among adolescents compared to children [3].

However, to the best of our knowledge, no multicenter study in Latin America has compared demographic features, clinical manifestations, laboratory findings, and treatments between IgAV/HSP children and adolescents at disease onset. Therefore, the objective of the present study was to compare demographic data, clinical and laboratory features, and treatments in children *versus* adolescents with IgAV/HSP within the first three months after diagnosis in a large multicenter Latin American population.

## Methods

A retrospective multicenter study included 687 children and adolescents (≤ 18 years-old) diagnosed with IgAV/HSP, followed-up in four pediatric rheumatology referral and university centers in Brazil. All participants met the validated classification criteria for IgAV/HSP established by the European League Against Rheumatism (EULAR), the Pediatric Rheumatology International Trials Organization (PRINTO), and the Pediatric Rheumatology European Society (PRES) [10, 11]. None of the patients had acute hemorrhagic edema of infancy (Finkelstein–Seidlmayer vasculitis) [12].

An online investigator meeting was held for this IgAV/HSP study to refine the protocol, which covered demographic and clinical data, treatment, and outcome parameters. A group of investigators supervised data collection at each center and resolved discrepancies through multiple rounds of queries to ensure accuracy. Data were systematically collected and managed using REDCap electronic data capture tools, over a period of 17 months, from December 2023 to April 2025.

The IgAV/HSP charts were retrospectively assessed for demographic data, clinical manifestations, laboratory findings, and therapeutic interventions at first 3 months after diagnosis. Ethical approval was obtained of all institutional review boards of participating centers.

Demographic data was composed by age at diagnosis, sex and period from symptom onset to diagnosis. Body mass index (BMI) was calculated according to weight in kilograms divided by height squared in meters (kg/m²). Potential triggers for IgAV/HSP, such as confirmed *Streptococcus pyogenes* infection, were also investigated.

Cutaneous involvement of IgAV/HSP included both recurrent and persistent purpura and/or petechiae. Recurrent purpura and/or petechiae were identified by the reappearance of lesions after complete resolution, whereas persistent lesions were defined as those lasting ≥ 6 weeks [13]. Arthritis was defined as joint edema or pain associated with restricted range of motion, while arthralgia was characterized to joint pain in the absence of swelling or limitation on movement [11, 13]. Recurrent arthritis and/or arthralgia were identified by the reappearance of these musculoskeletal involvements after complete resolution, whereas persistent arthritis and/or arthralgia were defined as those lasting ≥ 6 weeks [1, 13].

Abdominal pain of IgAV/HSP was considered as acute, diffuse, and colicky in nature. Severe abdominal pain was described by the presence of one or more of the following: abdominal angina, bowel intussusception and/or gastrointestinal bleeding. Recurrence of abdominal pain was described as the return of abdominal symptoms after its complete resolution. Abdominal Doppler ultrasonography was also used to evaluate patients with severe gastrointestinal involvement [1, 13].

IgAV/HSP nephritis was defined by the presence of one or more of the following abnormalities: hematuria (more than 5 red blood cells per high-power field and/or red blood cell count per mL above the laboratory reference range), proteinuria (greater than 0.1 g/m²/day or more than > 4 mg/m^2^/hour), cylindruria (presence of granular, red blood cell, and/or white blood cell casts), nephrotic syndrome and/or acute kidney injury (AKI) [1, 10]. Nephrotic syndrome was characterized as proteinuria greater than 1.0 g/m²/day or 50 mg/kg/day, typically associated with the presence of edema and serum albumin levels below 2.5 g/L [1]. Arterial hypertension was identified as IgAV patients with systolic and/or diastolic blood pressure at or above the 95th percentile for age, sex, and height, measured on three or more separate occasions [14].

AKI was defined as an increase in serum creatinine of ≥ 0.3 mg/dL (26.5 µmol/L) from baseline within 48 h, or an increase in serum creatinine to ≥ 1.5 times the baseline value within seven days, or urine output ≤ 0.5 mL/kg/hour for six hours, according to the definitions of Kidney Disease Improving Global Outcomes (KDIGO) criteria [15]. Chronic kidney disease (CKD) was identified by the presence of renal alterations persisting for at least three months, or by a sustained reduction in glomerular filtration rate less than 60 mL/min/1.73 m² over the same period [15]. Information on the indication and performance of renal biopsy, as well as the evaluation of renal replacement therapy (hemodialysis, peritoneal dialysis, hemofiltration, and/or kidney transplantation), was also recorded.

Orchitis/orchiepididymitis of IgAV/HSP was characterized by scrotal edema and pain or tenderness on physical examination and/or testicular abnormalities on Doppler ultrasonography [16, 17]. Neuropsychiatric involvement was defined as presence of at least one neurological manifestation, comprising isolated central nervous system vasculitis, headaches, seizures, coma, brain hemorrhage, Guillain-Barre syndrome, posterior reversible encephalopathy syndrome (PRES), ataxia, and/or central or peripheral neuropathy [18].

Pulmonary vasculitis of IgAV/HSP was defined as the presence of dyspnea, tachypnea, cough, and hemoptysis (probable pulmonary vasculitis), after exclusion of other vasculitis etiologies. Diffuse interstitial or alveolar infiltrates seen on chest radiography and/or high-resolution computed tomography (HRCT), abnormalities found in pulmonary function tests, and/or a reduced diffusing capacity for carbon monoxide (DLCO) defined confirmed IgAV/HSP with pulmonary vasculitis [4].

Reference ranges for laboratory parameters were set by each center’s clinical laboratory standards. Laboratory parameters assessed included serum IgA, IgG, complement levels, C-reactive protein (CRP), erythrocyte sedimentation rate (ESR), complete blood count, and urinalysis.

Treatments at the first three months of IgAV/HSP diagnosis included glucocorticosteroid (oral prednisone or prednisolone, and/or intravenous methylprednisolone), antiproteinuric agents (such as angiotensin-converting enzyme inhibitors), immunosuppressive agents [methotrexate, azathioprine, intravenous cyclophosphamide and cyclosporine], and intravenous immunoglobulin (IVIG).

Data were compared between IgAV/HSP children (< 10 years-old) and adolescents (≥ 10 years-old), according to World Health Organization (WHO) definition [19].

### Statistical analysis

Data were presented as median (minimum to maximum values) or mean ± standard deviation (SD) for continuous variables, and as frequency (percentage) for categorical variables. Comparisons between children and adolescents were made using the Mann-Whitney U test or Student’s t-test for continuous variables, and Fisher’s exact test for categorical variables. Logistic regression analysis models were done using adolescents (≥ 10 years-old) as a dependent variable and independent variables that presented a statistical significance level in the univariate analyses. A p-value < 0.05 was considered statistically significant.

## Results

Table 1 illustrates demographic and clinical manifestations in 687 with IgAV/HSP according to age at diagnosis. IgAV/HSP was diagnosed in 599/687 (87%) children [5.33(0.88–9.91) years-old] and 88/687 (13%) adolescents [11.33(10-17.5) years-old]. The median duration of purpura/petechiae was significantly lower in children compared to adolescents [14 (1-120) vs. 15 (2–90) days, *p* = 0.04]. The frequency of persistent purpura/petechiae was significantly reduced in the former group (7.2% vs. 19.5%, *p* = 0.002), likewise the frequency of gastrointestinal bleeding (17% vs. 34.1%, *p* = 0.01) and proteinuria (49.7% vs. 84%, *p* = 0.002). In contrast, the frequencies of arthritis/arthralgia (82.7% vs. 73%, *p* = 0.03) and orchitis (16.6% vs. 4.8%, *p* = 0.04) were significantly higher in children. The other demographic and clinical manifestations were similar in both groups (*p* > 0.05) (Table 1).

**Table 1:** Demographic and clinical manifestations in 687 patients with IgA vasculitis, formerly known as Henoch-Schönlein purpura (HSP), according to age at diagnosis

Variables at diagnosis *n* (%)	Children (< 10 years) (*n* = 599)	Adolescents (≥ 10 years) (*n* = 88)	*p*-value
**Demographic data**			
Age at diagnosis, years	5.33 (0.88–9.91)	11.33 (10-17.5)	**0.001**
Days between age of onset and age at diagnosis	7 (0-579)	10.0 (0-731)	0.33
Female sex	297 (49.6)	49 (55.7)	0.17
Body mass index, kg/m^2^	16.23 (10.8–31.0)	18.4 (12.6–31.7)	**0.001**
**Potential trigger**			
Confirmed *Pyogenes Streptococcal* infection	35/215 (16.3)	5/35 (14.3)	0.80
Benzathine penicillin prophylaxis	32/593 (5.4)	7/88 (8.0)	0.33
**Cutaneous/mucocutaneous involvement**			
Purpura	586/598 (98.0)	86/88 (98.0)	0.70
Petechiae	273/598 (45.7)	35/88 (39.8)	0.57
Number of episodes of purpura/petechiae	1 (1–4)	1 (1–4)	1.00
Purpura/petechiae duration, days	14 (1-120)	15 (2–90)	**0.04**
Persistent purpura/petechiae	42/587 (7.2)	17/87 (19.5)	**0.002**
Recurrent purpura/petechiae	91/574 (15.9)	9/85 (10.6)	0.42
**Articular involvement**			
Arthritis/arthralgia	494/597 (82.7)	64/88 (73.0)	**0.03**
Number of episodes of arthritis/arthralgia	1.0 (1–3)	1.0 (1–2)	0.63
Arthritis/arthralgia duration, days	7.0 (1-113)	7.0 (1-100)	0.40
Persistent arthritis/arthralgia	8/484 (1.7)	3/63 (4.8)	0.13
Recurrent arthritis/arthralgia	24/486 (4.9)	2/64 (3.1)	1.00
**Gastrointestinal involvement**			
Abdominal pain	373/598 (62.4)	46/88 (52.3)	0.08
Abdominal pain duration, days	7.0 (1–90)	9 (1–60)	0.21
Moderate to severe abdominal pain	151/314 (48.1)	19/40 (47.5)	0.87
Persistent abdominal pain	11/266 (3.0)	3/43 (7.0)	0.17
Recurrent abdominal pain	50/368 (13.6)	2/44 (4.5)	0.10
Gastrointestinal bleeding	63/371 (17.0)	15/44 (34.1)	**0.01**
Bowel intussusception	2/369 (0.5)	0 (0)	1.00
**Renal involvement**	170/598 (28.4)	27/88 (30.7)	0.53
Systemic arterial hypertension	33/448 (7.4)	5/74 (6.8)	1.00
Nephrotic syndrome	10/79 (12.7)	4/21 (19.0)	0.50
Hematuria	135/167 (81.0)	21/27 (78.0)	0.44
Leukocyturia	72/165 (43.3)	5/24 (21.0)	**0.04**
Urinary casts	8/163 (4.9)	2/24 (8.0)	0.62
Proteinuria	84/168 (49.7)	21/25 (84.0)	**0.002**
Acute kidney injury	28/167 (16.8)	4/25 (16.0)	1.00
Chronic kidney disease	1/165 (0.6)	0 (0)	1.00
Persistent renal involvement	55/167 (33.0)	9/25 (36.0)	1.00
Recurrent renal involvement	16/167 (9.6)	2/25 (8.0)	1.00
**Genital involvement**			
Orchitis	55/331 (16.6)	2/42 (4.8)	**0.04**
Episodes of orchitis	1 (1–3)	1 (1–1)	0.51
Orchitis duration, days	5 (1–20)	3 (3–3)	0.47
**Neuropsychiatric involvement**	1/598 (0.2)	1/88 (1.1)	0.24
**Pulmonary involvement**	1/598 (0.2)	0 (0)	1.00

Results are presented as median (minimum-maximum value) or *n* (%)

Table 2 shows laboratory findings and treatments in 687 patients with IgAV/HSP according to age at diagnosis. The median value of serum IgA was significantly lower in children than in adolescents [179.1 (40-1002.0) vs. 279.0(104.0-488.0) mg/dL, *p* = 0.01], whereas thrombocytosis was significantly higher in the children (40.1% vs. 23%, *p* = 0.007). No differences were evidenced regarding other laboratory parameters and treatments in both groups (*p* > 0.05) (Table 2).

**Table 2:** Laboratory findings and treatments in 687 patients with IgA vasculitis, formerly known as Henoch-Schönlein purpura (HSP), according to age at diagnosis

Variables at diagnosis *n* (%)	Children (< 10 years) (*n* = 599)	Adolescents (≥ 10 years) (*n* = 88)	*p*-value
**Laboratory abnormalities**			
Serum IgA, UI/mL	179.1 (40-1002.0)	279.0 (104.0-488.0)	**0.01**
Increased serum IgA, UI/mL	38/141 (27.0)	5/17 (29.3)	0.70
Serum IgG, mg/dL	1110.0 (120–3409.0)	893.0 (189–1561.0)	0.15
Increased serum IgG, mg/dL	14/106 (13.2)	0 (0)	1.00
Serum C3, mg/dL	123.0 (0.5–216.0)	129.0 (2.0-1520.0)	0.61
Increased ESR, mm/1st hour	204/408 (50.0)	25/57 (43.9)	0.33
Increased CRP, mg/dL	169/371 (45.6)	22/51 (43.1)	0.55
Hemoglobin, g/dL	12.4 (6.7–17.7)	12.8 (9.8–17.1)	0.09
Thrombocytosis > 400.000/mm^3^	240/599 (40.1)	20/88 (23.0)	**0.007**
**Treatments**			
Oral glucocorticoid	309/594 (51.7)	47/87 (54.0)	0.82
Methotrexate	1/590 (0.2)	1/88 (1.1)	0.25
Azathioprine	5/589 (0.8)	0 (0)	1.00
Intravenous cyclophosphamide	1/590 (0.2)	1/86 (1.1)	0.24
Cyclosporine	0 (0)	1/86 (1.1)	1.00
Antiproteinuric	25/591 (4.2)	6/86 (6.8)	0.27
Antiproteinuric duration, days	54.0 (21.0-180.0)	210.0 (54-1095.0)	0.11

Results are presented as median (minimum - maximum value) or *n* (%), ESR - erythrocyte sedimentation rate, CRP - C-reactive protein, increased serum IgA (> 255 UI/mL), increased serum IgG (> 1822 mg/dL), increased ESR (> 20 mm/1st hour), increased CRP (> 5 mg/L)

A logistic regression analysis was performed, including seven independent variables that reached statistical significance (*p* < 0.05) in univariate analysis: persistent purpura/petechiae, arthritis/arthralgia, gastrointestinal bleeding, orchitis, leukocyturia, proteinuria, and thrombocytosis. This model showed that persistent purpura/petechiae after IgAV/HSP diagnosis (OR = 18.337; 95% CI 1.245-270.137; *p* = 0.034) was the only independently variable associated with adolescents.

## Discussion

Our Brazilian multicenter cohort study found that IgAV/HSP was rare in adolescence and often presented with a more prominent cutaneous involvement in this age group. Prolonged purpura/petechiae following IgAV/HSP diagnosis was associated with adolescent-onset.

This research has advantages. The multicenter study included analysis of IgAV/HSP patients followed up at four university and tertiary centers in a Latin America, and all patients fulfilled the validated criteria of EULAR/PRINTO/PRES for IgAV/HSP [10]. We also excluded patients with acute hemorrhagic edema, as a recent systematic review showed that only a quarter of these cases had documented IgA deposits in skin biopsy immunofluorescence, suggesting different disease underlying mechanisms between acute hemorrhagic edema and IgAV [12].

In a large population of IgAV/HSP patients, we extended previous findings and showed that persistent purpura/petechiae after diagnosis occurred more frequently in adolescents than in children. Approximately 20% of our adolescents with IgAV/HSP had persistent skin manifestations, a rate notably higher than the 7.4% reported in a recent study of 450 patients with a median age at diagnosis of 7.25 years [5]. In addition, persistent or recurrent disease was described in a small case series of 13 atypical IgAV/HSP patients—particularly in older children—which may lead to a more severe course and increased disease burden [6]. Another study also linked persistent or relapsing purpura to IgAV/HSP renal involvement, a serious manifestation associated with higher morbidity and mortality [5, 20].

Moreover, our findings indicated a more severe presentation of IgAV/HSP in adolescents, particularly with proteinuria and gastrointestinal bleeding. Consistent with this, a Turkish study reported higher rates of nephritis and more frequent use of aggressive treatments in adolescents compared to children [3]. A meta-analysis on risk factors for nephritis also found that adolescent age, compared to younger children, was associated with this complication [20].

The higher IgA levels observed in our adolescents highlight potential immunopathological differences between age brackets. Prior literature has also reported elevated IgA levels in IgAV/HSP patients with nephritis [21].

On the other hand, children more frequently experience arthritis, arthralgia, and orchitis at diagnosis herein, suggesting a milder to moderate disease compared to adolescents. The musculoskeletal findings (arthritis and/or arthralgia) contrast with Coşkun et al. [3], that showed higher frequency of this involvement in adolescents than in younger children. This discrepancy between these two age groups in Brazilian and Turkish reports may be related to genetic background, referral patterns, and timing of clinical evaluation. Further research will be necessary to elucidate these observations.

The retrospective design was the major limitation of the present study. However, the use of a standardized database and investigator supervision at each center, along with multiple query rounds to ensure data accuracy, helped to reduce this methodological weakness and potential bias. Another limitation was the lack of assessment of self-reported ethnicity and race. Although all four participating pediatric rheumatology referral centers are located in large urban areas in Brazil, we did not evaluate the geographical origin of each patient, which represents an additional limitation.

## Conclusions

In this large multicenter cohort showed that IgAV/HSP onset occurred rarely at adolescence, with a more severe disease. The persistence of purpura/petechiae after an IgAV/HSP diagnosis was associated with adolescent-onset, underscoring the importance of vigilant monitoring in this patient subgroup.

## Data Availability

All data generated or analyzed during this study are included in this published article [and its supplementary information files].

## Abbreviations

BMI: Body mass index
CKD: Chronic kidney disease
CRP: C–reactive protein
DLCO: Diffusing capacity for carbon monoxide
EULAR: European League Against Rheumatism
ESR: Erythrocyte sedimentation rate
HSP: Henoch–Schönlein purpura
HRCT: Resolution computed tomography
IgAV: Immunoglobulin A vasculitis
IVIG: Immunoglobulin
KDIGO: Kidney Disease Improving Global Outcomes
PRES: Pediatric Rheumatology European Society
PRINTO: Pediatric Rheumatology International Trials Organization
PRES: Posterior reversible encephalopathy syndrome
SD: Standard deviation
WHO: World Health Organization
